# The Bag-1 inhibitor, Thio-2, reverses an atypical 3D morphology driven by Bag-1L overexpression in a MCF-10A model of ductal carcinoma *in situ*

**DOI:** 10.1038/oncsis.2016.10

**Published:** 2016-04-04

**Authors:** E S Papadakis, C R Barker, H Syed, T Reeves, S Schwaiger, H Stuppner, J Troppmair, J P Blaydes, R I Cutress

**Affiliations:** 1 Cancer Research UK Centre Cancer Sciences Unit, University of Southampton Faculty of Medicine, Southampton General Hospital, Southampton, UK; 2 Institute of Pharmacy/Pharmacognosy, Center of Molecular Biosciences, University of Innsbruck, Innrain, Innsbruck, Austria; 3 Daniel Swarovski Research Laboratory, Department of Visceral-, Transplant- and Thoracic Surgery, Innsbruck Medical University, Austria; 4 University Hospital Southampton, University of Southampton Faculty of Medicine, Southampton General Hospital, Southampton, UK

## Abstract

Mammary MCF-10A cells seeded on reconstituted basement membrane form spherical structures with a hollow central lumen, termed acini, which are a physiologically relevant model of mammary morphogenesis. Bcl-2-associated athanogene 1 (Bag-1) is a multifunctional protein overexpressed in breast cancer and ductal carcinoma *in situ*. When present in the nucleus Bag-1 is predictive of clinical outcome in breast cancer. Bag-1 exists as three main isoforms, which are produced by alternative translation initiation from a single mRNA. The long isoform of Bag-1, Bag-1L, contains a nuclear localisation sequence not present in the other isoforms. When present in the nucleus Bag-1L, but not the other Bag-1 isoforms, can interact with and modulate the activities of estrogen-, androgen- and vitamin D-receptors. Overexpression of Bag-1 mRNA in MCF-10A is known to produce acini with luminal filling reminiscent of ductal carcinoma *in situ*. As this mRNA predominantly overexpresses the short isoform of Bag-1, Bag-1S, we set out to examine whether the nuclear Bag-1L isoform is sufficient to drive premalignant change by developing a Bag-1L-overexpressing MCF-10A model. Two clones differentially overexpressing Bag-1L were grown in two-dimensional (2D) and three-dimensional (3D) cultures and compared with an established model of HER2-driven transformation. In 2D cultures, Bag-1L overexpression reduced proliferation but did not affect growth factor responsiveness or clonogenicity. Acini formed by Bag-1L-overexpressing cells exhibited reduced luminal clearing when compared with controls. An abnormal branching morphology was also observed which correlated with the level of Bag-1L overexpression, suggesting further malignant change. Treatment with Thio-2, a small-molecule inhibitor of Bag-1, reduced the level of branching. In summary, 3D cultures of MCF-10A mammary epithelial cells overexpressing Bag-1L demonstrate a premalignant phenotype with features of ductal carcinoma *in situ*. Using this model to test the small-molecule Bag-1 inhibitor, Thio-2, reveals its potential to reverse the atypical branched morphology of acini that characterizes this premalignant change.

## Introduction

Investigation of breast cancer progression has traditionally relied on two-dimensional (2D) *in vitro* cell models to elucidate the molecular drivers associated with this disease. However, 2D culture has spatial and cell differentiation limitations.^1, 2^ Three-dimensional (3D) cell models provide an important additional experimental tool as they recapitulate several aspects of normal and tumour tissue architecture. When seeded in reconstituted basement membrane (BM), human mammary epithelial normal cells and non-tumourigenic cell lines, such as MCF-10A, follow a highly regulated morphogenetic program to form organized growth-arrested 3D spherical structures with a central lumen termed acini.^3, 4^ This involves polarization of BM-attached outer cells with concomitant anoikis and metabolic changes of luminal cells that are no longer in contact with the BM (BM-detached).^5, 6^ Expression analysis of acini morphogenesis has led to the identification of a 3D molecular signature, comprising genes that are downregulated during this process, which accurately predicts outcome in breast cancer patients.^7, 8^

Contrary to normal breast cells, breast tumour cells and breast cancer cell lines continue to proliferate and give rise to larger acini with altered external morphology and no central lumen owing to defects in anoikis and metabolism of BM-detached cells;^2, 3, 5, 9^ these structures bear features of ductal carcinoma *in situ*, a precursor to invasive ductal carcinoma.^10^ Comparison of the transcriptional profiles of a panel of human breast cancer cell lines correlates with their 3D morphology and enables clustering into four main phenotypic subclasses, which are associated with clinically distinct subgroups.^2^ The majority of basal B subtype cell lines form invasive stellate structures, which lack E-cadherin and EGFR/HER2 expression, whereas luminal and basal A subtypes form round, mass, or grape-like structures, which often overexpress HER2, a marker of poor prognosis in breast cancer.^2, 11^ Consistent with this, HER2 overexpression or activation in MCF-10A cells results in atypical grape-like acinar morphology and impedes lumen formation by attenuating apoptosis and by driving metabolic changes in BM-detached cells.^5, 9, 12, 13, 14^ Culture of MCF-10A cells in 3D therefore constitutes a powerful tool for the study of oncogene-driven premalignant changes.

The co-chaperone protein Bcl-2-associated athanogene 1 (Bag-1) is expressed at low levels in most human tissues^15^ but is frequently overexpressed in invasive breast carcinoma and importantly also within the preinvasive ductal carcinoma *in situ* stage.^15, 16, 17, 18, 19^ In the clinical setting of breast cancer, Bag-1 mRNA is a prognostic biomarker included within the Oncotype DX and PAM50 multigene assays^20, 21^ and high nuclear Bag-1 immunoreactivity is an independent predictor of outcome and enhances the predictive power of IHC4 score (a combination of the prognostic information from ER, PgR, Ki67 and HER2 immunohistochemical staining).^16, 22, 23, 24, 25^ At the cellular level, Bag-1 interacts with a number of protein partners including Bcl-2, Hsc70/Hsp70 chaperones, and nuclear hormone receptors to promote cell survival.^26^ Proof-of-principle studies from our laboratory have shown that it is possible to restrict breast cancer and melanoma cell growth by targeting Bag-1 protein–protein interactions using synthetic peptides and small-molecule compounds like Thioflavin S and its biologically active constituent Thio-2.^27, 28, 29^ Bag-1 exists as three main isoforms Bag-1S and Bag-1M, which are mainly localized in the cytoplasm, and Bag-1L, which contains a nuclear localisation signal not present in the other isoforms and is predominantly localized in the nucleus.^15, 19, 30, 31, 32, 33^ Bag-1L, but not Bag-1M or Bag-1S, enhances the transcriptional activity of androgen receptor,^32, 34, 35^ vitamin D receptor,^36^ and oestrogen receptor.^22^ Xenograft studies show that Bag-1L overexpression drives growth of breast tumours formed by oestrogen-responsive ZR-75-1 breast cancer cells in an oestrogen-dependent manner.^37^ Although clinical studies and mouse models have shed some light on the role of Bag-1L in breast cancer pathology, little is known about the role of Bag-1L in initiating premalignant change in the breast.

Studies using a 3D cell culture model have shown that concomitant co-overexpression of the main Bag-1 isoforms (Bag-1S, Bag-1M and Bag-1L) in MCF-10A cells leads to the formation of lumenless acini, through attenuation of anoikis in BM-detached cells, without the grape-like abnormalities in morphology seen with HER2 overexpression.^38^ However, there is currently no evidence on the ability of individual Bag-1 isoforms to regulate acinar morphology. Based on published data supporting an important role of nuclear Bag-1L in breast cancer, in this study we sought to examine the effect of the individual Bag-1L isoform on acini morphogenesis to elucidate and describe its role in promoting premalignant change in 3D. We then examined the effect of a small-molecule inhibitor of Bag-1, Thio-2, in Bag-1L-driven premalignant change in this experimental model of ductal carcinoma *in situ* to determine whether these changes might be amenable to therapeutic intervention.

## Results

### Characterization of Bag-1L overexpression in 2D culture

**Figure 1:**
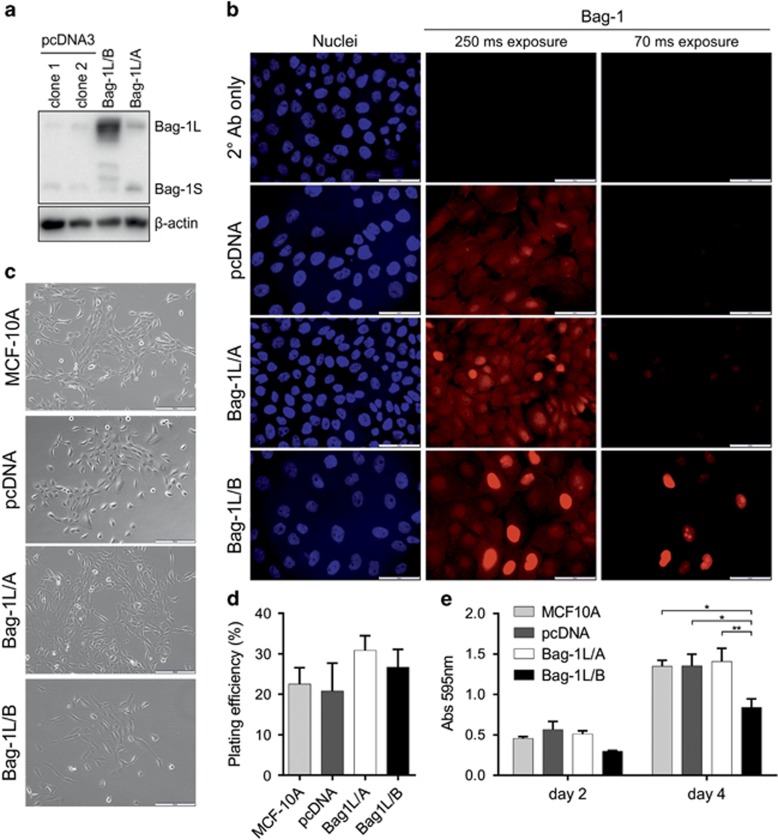
Effect of Bag-1L overexpression on MCF-10A 2D cultures. Bag-1 protein expression was examined in MCF-10A clones grown in 2D cultures as indicated by: (**a**) Immunoblot where β-actin was included as a control for loading and (**b**) immunocytochemical labeling for Bag-1 (red) and nuclear counterstain DAPI (blue); two exposure times are shown for Bag-1 staining; scale bars=50 μm. (**c**) Phase-contrast images reveal that Bag-1L-overexpressing clones acquire a cobblestone morphology, which is typical of MCF-10A cells; scale bars=200 μm. (**d**) The colony-forming efficiency of Bag-1L clones was assessed in a clonogenic assay and expressed as the plating efficiency. Values represent the mean±s.e.m. from three independent experiments, each with three technical replicates. (**e**) Proliferation of clones was measured at days 2 and 4 of culture. Cells (20 000/well) were plated at day 0 and were fixed in methanol and stained with crystal violet. Stain was dissolved in 20% acetic acid and absorbance at 595 nm recorded. Bar graphs represent the mean±s.e.m. values from three independent experiments, each with three technical replicates. *P<0.05, **P<0.01 as determined by two-way ANOVA with Bonferroni's multiple comparisons test.

To examine the potential role of Bag-1L in breast tumourigenesis, MCF-10A stable cell clones were generated by transfection of a pcDNA3 vector containing Bag-1 complementary DNA with an optimized Bag-1L start site.^22^ Immunoblot analysis revealed the presence of two clones overexpressing Bag-1L at low (Bag-1L/A) and high (Bag-1L/B) levels compared with two clones containing empty pcDNA3 vector of which clone 1, designated pcDNA, was used as a control for this study (Figure 1a). Immunofluorescence staining revealed higher but heterogeneous expression of Bag-1 in the nucleus of both clones compared with pcDNA and was more intense in Bag-1L/B (Figure 1b), which is consistent with overexpression of Bag-1L.

To characterize the effect of Bag-1L overexpression in 2D culture, cell morphology, colony-forming efficiency and proliferation were examined. Cell clones assumed a cobblestone appearance typically observed in parental MCF-10A cells with lamellipodia extending from the edges of clusters^4^ (Figure 1c). Bag-1L overexpression did not alter the colony-forming efficiency of MCF-10A cells (Figure 1d) and supported neither anchorage-independent growth *in vitro* nor tumour growth *in vivo* (data not shown). Moreover, Bag-1L/B cells displayed a significant decrease in proliferation compared with pcDNA, as shown by a 38% reduction in crystal violet absorbance, whereas Bag-1L/A cells proliferated comparably to pcDNA controls (Figure 1e).

### Effect of Bag-1L overexpression on acini morphogenesis

**Figure 2:**
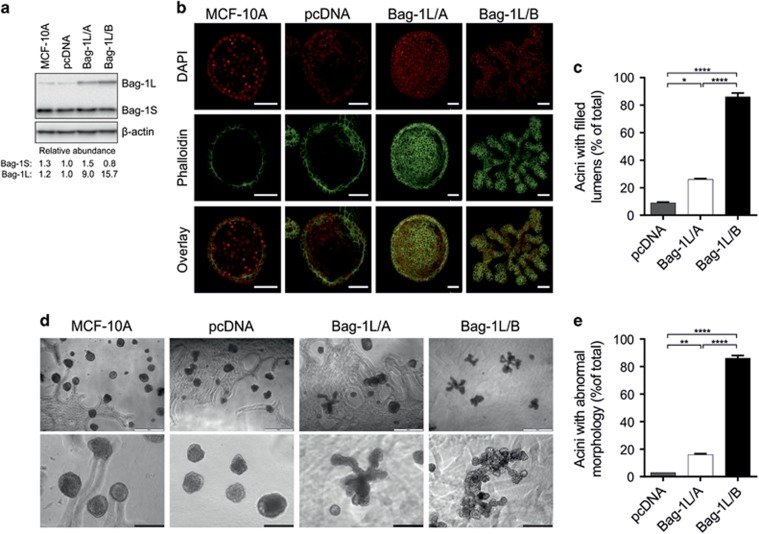
Bag-1L overexpression attenuates luminal clearing and promotes an abnormal acinar morphology. (**a**) Immunoblot analysis shows expression of Bag-1 isoforms in lysates from MCF-10A acini clones cultured for 12 days; β-actin was included as a loading control. Densitometric analysis shows the abundance of Bag-1 S and Bag-1L protein isoforms relative to pcDNA control. (**b**) Representative confocal immunofluorescence images taken through the center of MCF-10A acini at day 20 of culture. Cells were stained with phalloidin-TRITC (green) and nuclei counterstained with DAPI (red); scale bars=100 μm. (**c**) Acini with filled lumens were counted at day 20 of culture and their number is expressed as a percentage of total acini. (**d**) Representative phase-contrast images of MCF-10A cell clones cultured in 3D for 20 days revealing gross external morphology; white scale bars=500 μm; black scale bars=200 μm. (**e**) Acini with abnormal morphology were counted at day 20 of culture and their number is expressed as a percentage of total acini. Values represent the mean±s.e.m. from at least three independent experiments, each with two technical replicates. *P<0.05, **P<0.01, ****P<0.0001 as determined by two-way ANOVA with Bonferroni's multiple comparisons test.

MCF-10A acini provide a physiologically relevant model to study the influence of Bag-1L overexpression on 3D morphology. Compared with pcDNA acini, Bag-1L protein level was ninefold higher in Bag-1L/A and 16-fold higher in Bag-1L/B, thereby providing a basis for the study of Bag-1L in acini morphogenesis (Figure 2a). Confocal microscopy images at day 20 of culture indicated formation of a central lumen in MCF-10A parental and pcDNA acini as expected (Figure 2b). In contrast, 26% of Bag-1L/A and 86% of Bag-1L/B acini were lumenless compared to 9% lumenless acini formed by pcDNA cells (Figures 2b and c). Moreover, phase-contrast microscopical examination revealed that 16% of Bag-1L/A and 86% of Bag-1L/B acini assumed a branched morphology (Figures 2d and e), which resembled an atypical phenotype in breast cancer cells reported by Kenny *et al.*^2^ Quantification of confocal and phase-contrast data indicated that the increase in Bag-1L expression observed between Bag-1L/A and Bag-1L/B clones is associated with a significant 3.3-fold increase in the number of atypical acini formed with no central lumen (Figure 2c) and with altered external morphology (Figure 2e).

**Figure 3:**
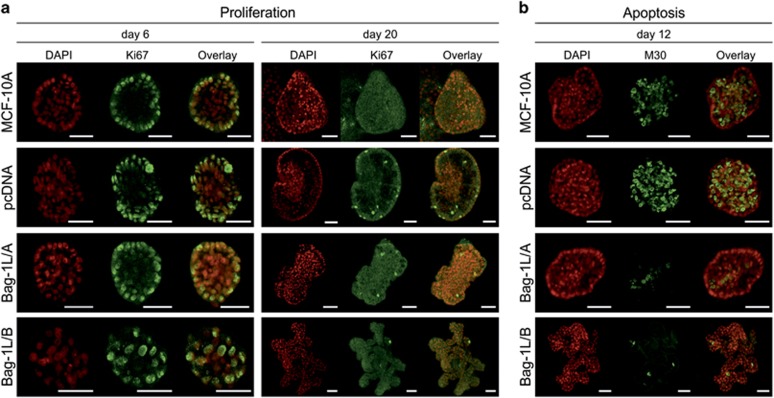
Bag-1L overexpression does not prevent cell cycle arrest but attenuates luminal apoptosis during acini morphogenesis. Representative confocal immunofluorescence images taken through the center of MCF-10A acini at the indicated time points of 3D culture. (**a**) Proliferation was examined using Ki67 (green) as a marker. (**b**) Apoptosis was assessed using M30 (green) as a marker of caspase-cleaved cytokeratin 18. Nuclei were counterstained with DAPI (red); scale bars=50 μm.

To further characterize the effect of Bag-1L overexpression on acinar phenotype, cell proliferation and apoptosis were examined by immunofluorescence staining (Figure 3). At day 6 of culture, Ki67, a protein marker of proliferation,^39^ was present in BM-attached cells situated at the periphery of control and Bag-1L/A-overexpressing acini, whereas in Bag-1L/B-overexpressing acini it was present throughout (Figure 3a). At day 20 of culture, cells in control and Bag-1L-overexpressing acini exhibited minimal Ki67 staining consistent with the induction of growth arrest (Figure 3a). In addition, luminal apoptosis that was detected by M30 (antibody recognising caspase-cleaved cytokeratin 18) was markedly increased in parental and pcDNA acini but was almost undetectable in Bag-1L/A and Bag-1L/B acini (Figure 3b) at day 10 of culture. Taken together, these data show that overexpression of the Bag-1L isoform promotes an atypical acinar phenotype as it impedes formation of a central lumen by maintaining proliferation and attenuating apoptosis, and induces a branched acinar morphology.

### Effect of HER2 overexpression on acini morphogenesis and comparison of 2D growth response with growth factors between Bag-1L- and HER2-overexpressing MCF-10A cells

**Figure 4:**
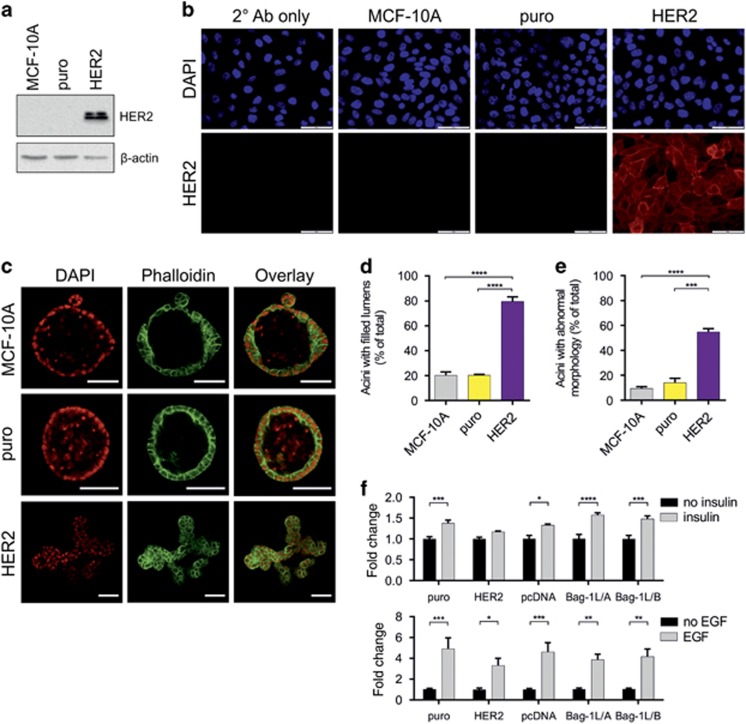
HER2 overexpression promotes atypical MCF-10A morphology in 3D and insulin unresponsiveness in 2D cultures. (**a**) Immunoblot analysis of HER2 overexpression in retrovirally transduced MCF-10A-pooled clones grown in 2D culture; β-actin was included as a control for loading. (**b**) Immunofluorescence staining for HER2 (red) in 2D cultures of MCF-10A parental or retrovirally transduced with pBabe-puro vector control (puro) or pBabe-puro/(HER2); nuclei were counterstained with DAPI (blue), whereas secondary antibody alone was used to exclude non-specific staining; scale bars=50 μm. (**c**) Representative confocal immunofluorescence images of MCF-10A acini at day 12 of culture. Acini were stained with phalloidin-TRITC (green) and nuclei counterstained with DAPI (red); scale bars=50 μm. The number of acini with (**d**) filled lumens (day 12) or (**e**) abnormal morphology (day 20) was counted and is expressed as a percentage of the total number of acini. Values represent the mean±s.e.m. from three independent experiments, each with four technical replicates. **P<0.01, ****P<0.0001 as determined by two-way ANOVA with Bonferroni's multiple comparisons test. (**f**) Insulin and EGF sensitivity was assessed in 2D culture under serum-free conditions (24 h serum starvation) following treatment with the indicated growth factors for 48 h. Bar graphs represent the mean fold change±s.e.m. in absorbance (595 nm), corresponding to cell growth, in growth factor-supplemented relative to growth factor-free media for each cell line, which was determined by crystal violet assay. Data are from at least three independent experiments, each with three technical replicates. *P<0.05, **P<0.01, ***P<0.001, ****P<0.0001 as determined by two-way ANOVA with Bonferroni's multiple comparisons test.

We next compared the 3D phenotypic changes observed in response to Bag-1L overexpression with a described and recognized^9, 13^ 3D model of HER2 transformation. HER2 protein overexpression was confirmed by immunoblotting (Figure 4a) and immunofluorescence staining (Figure 4b). Lumen formation in acini grown to day 12 and stained with phalloidin was examined by confocal microscopy (Figures 4c and d) and external morphology quantified from high-resolution phase-contrast microscopy images (Figure 4e). As expected, parental and pBABE-puro vector control puro cells (puro) gave rise to similar numbers of acini with a central lumen and a typical spherical structure (Figures 4c and e). In contrast, HER2 overexpression resulted in a fourfold increase in acini lacking a central lumen (Figure 4d) and a threefold increase in acini exhibiting branched morphology (Figure 4e) compared with puro control, in line with previous studies.^9, 13^ This 3D morphology was similar to that of Bag-1L-overexpressing clones.

Growth of MCF-10A cells requires insulin-like growth factor 1,^40^ which is chemically and functionally similar to insulin and facilitates cellular glucose uptake.^41^ Overexpression of HER2 causes metabolic transformation of MCF-10A cells, which is characterized by insulin-independent proliferation, and enhances glucose uptake in the absence of insulin-like growth factor 1 receptor activity.^5, 42, 43^ Based on the phenotypic similarities between HER2- and Bag-1L-overexpressing MCF-10A cells in 3D, we investigated whether the atypical phenotype of Bag-1L-overexpressing MCF-10A clones could also be due to metabolic changes associated with loss of responsiveness to insulin. To this end, cells were cultured in insulin-free or insulin-containing media using HER2-overexpressing MCF-10A clones as a control. In line with previous studies,^42^ no significant difference in the growth of HER2-overexpressing clones was observed under these conditions (Figure 4f). Conversely, growth of puro and pcDNA controls displayed a significant increase of ~1.4-fold in the presence compared with the absence of insulin (Figure 4f). Similarly, a growth increase of ~1.6-fold in Bag-1L/A and ~1.5-fold in Bag-1L/B was observed in the presence compared with the absence of insulin, indicating responsiveness to this hormone. Moreover, all cell lines were responsive to epidermal growth factor for growth (Figure 4f), consistent with previous reports.^43^ These data suggest that the mechanism responsible for the morphological changes observed in response to Bag-1L overexpression is likely to be different to that of HER2 and requires further investigation.

### Effect of Bag-1 inhibitors on 2D culture and acini morphogenesis

**Figure 5:**
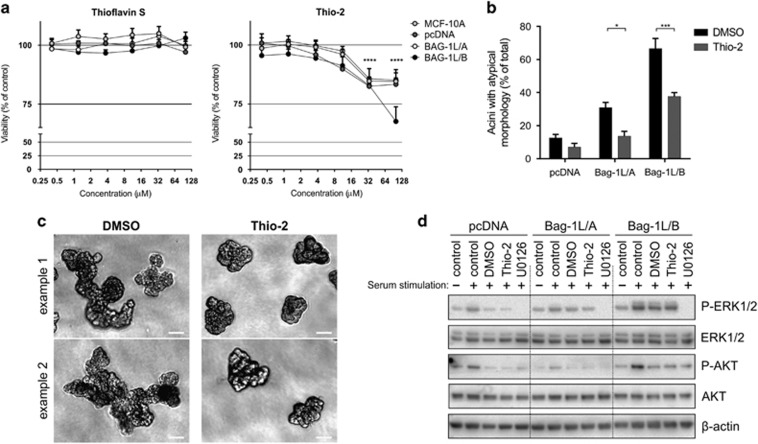
Thio-2 can partially reverse the abnormal acinar morphology associated with Bag-1L overexpression. (**a**) MCF-10A cells were treated with Thioflavin S or Thio-2 at the indicated concentrations for 5 days and viability was assessed by CellTiter Aqueous One solution assay relative to DMSO-treated cells at each concentration. Values represent the mean±s.d. as percentage of DMSO control from three experiments, each with three technical replicates. ****P<0.0001 was determined by two-way ANOVA with Bonferroni's multiple comparisons test relative to the effect of the lowest concentration of Thio-2 on each corresponding cell type. (**b**) MCF-10A cells were treated for 24 h with Thio-2 (50 μM) or DMSO (0.5% v/v) before seeding on Matrigel and acini were allowed to form over 14 days; further treatment (25 μM Thio-2 or 0.25% v/v DMSO) was administered on days 4 and 8 of 3D culture. Acini exhibiting abnormal morphology were quantified and expressed as a percentage of the total number. Values represent the mean±s.e.m. from four independent experiments, each with two technical replicates. *P<0.05, **P<0.01, ***P<0.001 as determined by two-way ANOVA with Bonferroni's multiple comparisons test. (**c**) Representative phase-contrast microscopy images show the atypical branching external morphology of Bag-1L/B acini in the presence of DMSO or Thio-2; scale bars=50 μm. (**d**) Immunoblot analysis shows the effect of serum (10%) stimulation alone or in the presence of DMSO (0.5%), Thio-2 (50 μM) or U0126 (25 μM) on the activation of ERK and AKT in serum-deprived cells; β-actin was used as a loading control.

MCF-10A clones were examined in the presence of Thioflavin S and Thio-2 inhibitors of Bag-1 protein–protein interactions.^28, 44^ Viability assay data show that Thio-2 significantly decreased growth across all cell lines at 50 μM and 100 μM, whereas Thioflavin S had no effect (Figure 5a), confirming our previously published findings.^44^ Therefore, Thio-2 was used in subsequent experiments.

We examined whether the atypical morphology of Bag-1L-overexpressing acini could be pharmacologically reversed. MCF-10A cells grown in 2D were pre-treated with Thio-2 for 24 h before seeding on Matrigel with additional treatments administered on days 4 and 8 of 3D culture. Thio-2 treatment significantly reduced the number of atypical acini by 17% in Bag-1L/A and 29% in Bag-1L/B compared with dimethyl sulfoxide control (Figure 5b). Acini displaying atypical external morphology exhibited a reduced level of branching (Figure 5c), suggesting that changes in morphology could be due to Bag-1 protein–protein interactions. Furthermore, there was no noticeable inhibitory effect by Thio-2 treatment on the activities of ERK and AKT as shown by immunoblot analysis (Figure 5d), implying that these signalling pathways may not be involved in the atypical branching phenotype.

## Discussion

Our data demonstrate, for the first time, that overexpression of the Bag-1L isoform alone is sufficient to suppress luminal apoptosis and drives the formation of MCF-10A acini with a branched morphology, which resemble transformed acini formed by HER2-overexpressing MCF-10A cells, generated here by us and published by others.^9, 13^ Although studies by Anderson *et al.*^38^ revealed the existence of reduced luminal clearing in acini co-overexpressing all three main Bag-1 isoforms (Bag-1S, Bag-1M, Bag-1L) no observation of abnormal branched morphology was reported. Similar to previous observations in non-tumourigenic HaCaT skin epidermal keratinocytes,^45^ our data also show that Bag-1L overexpression results in decreased MCF-10A cell growth in 2D cultures, an event that seems to be inversely correlated to its level of expression.

MCF-10A acini morphogenesis proceeds through a highly regulated sequence of events. Cell detachment from the BM in the lumen of acini induces cellular stresses resulting in anoikis and luminal clearing. This occurs through downregulation of ERK activity, resulting in potentiation of the pro-apoptotic activity of Bim_EL_.^1, 4, 6, 12^ Studies by Anderson *et al.*^38^ in which Bag-1S, Bag-1M and Bag-1L isoforms were co-expressed in MCF-10A cells reveal that this process can be impeded by targeting the pro-apoptotic protein Bim_EL_ for proteasomal degradation through enhanced activation of the RAF-1/MEK/ERK signalling cascade. Although we have previously shown that Thio-2 downregulates the activity of ERK in MCF7 cells,^44^ this was not the case in this MCF-10A model. Studies by Debnath *et al.*^6^ have shown that apoptosis inhibition alone is insufficient to prevent luminal clearing, with Bcl-2 overexpression delaying but not completely preventing anoikis; this has implicated the existence of an additional metabolic mechanism involving the upregulation of cellular antioxidant defences.^5, 46^ To investigate the possibility that Bag-1L overexpression may suppress the metabolic impairments associated with detachment from the BM we examined insulin-stimulated growth based on the phenotypic resemblance observed between Bag-1L- and HER2-overexpressing acini. Although HER2 upregulation enhances glucose uptake independently of insulin-like growth factor 1 or insulin, leading to insulin insensitivity,^42^ Bag-1L-overexpression retained insulin responsiveness, implying dependence on insulin for glucose uptake. This suggests that despite the phenotypic similarities, Bag-1L-overexpressing acini can overcome the metabolic defects associated with detachment via a different transforming mechanism to HER2.

Targeting Bag-1 with its protein–protein interaction inhibitor Thio-2 attenuated the atypical branching of acini, suggesting that Bag-1L protein–protein interactions are important for determining the morphology displayed upon cell attachment to the BM. Importantly, the level of Bag-1L overexpression correlated with the phenotypic effect of compound treatment as Bag-1L/B clone exhibited the greatest reversal in morphology in response to treatment with Thio-2, thereby providing further evidence of a Bag-1L-driven phenotype. Although Anderson *et al.*^38^ described an inability of acini co-overexpressing Bag-1S, Bag-1M and Bag-1L to form a lumen, they did not report formation of a branched external morphology. This could be attributed to the regulatory balance exerted by the combined activities of individual Bag-1 isoforms on cell-to-cell contact. Evidence for this comes from studies by Hinnit *et al.*^45^ who measured single-cell movement from small colonies of HGF-stimulated HaCaT cells using scatter assays. They show that constitutive Bag-1L overexpression results in greater than twofold cell-to-cell dissociation, whereas Bag-1S and Bag-1M retain their cell-to-cell contacts and exhibit no scattering. When considered together with findings by Hinnit *et al.* and Anderson *et al.*, our data emphasize the importance of the combined activities of distinct Bag-1 isoforms on cellular function, and support a role for Bag-1L in the formation of a premalignant phenotype that might potentially be amenable to therapeutic intervention.

In summary, our data describe a role for the Bag-1L isoform as a driver of phenotypic changes associated with a premalignant state in the breast using a 3D model of ductal carcinoma *in situ*. Such changes seen in 3D models highlight the efficacy of these models to test the effects of inhibitors of breast tumour initiation in 3D that may not be seen in 2D. The ability of Thio-2 to reverse some of these changes highlights the potential of Bag-1L-overexpressing MCF-10A acini as a model to test the effect of inhibitors of breast tumour initiation.

## Materials and methods

### Cell culture and generation of stable clones

MCF-10A cells were obtained from LGC Standards (Teddington, UK) and were cultured and maintained as described by Debnath *et al.*^4^ To generate cell clones, pcDNA3-Bag-1L or empty pcDNA3 vectors were transfected into MCF-10A cells using FuGene HD (Promega, Southampton, UK) and stable integrants selected with Geneticin (100 μg/ml; Sigma, Gillingham, UK). Single clones were harvested by trypsinisation using cloning rings and expanded. HER2 clones were generated as described by Debnath *et al.*^4^ In brief, MCF-10A cells were infected using 0.45 μm filtered viral supernatant derived from Phoenix HEK-293 cells transfected with either pBABEpuro/HER2 vector^47^ (Addgene plasmid 40978) or with empty pBABE-puro vector (puro) as control, and were selected with puromycin (0.5 μg/ml). Resistant HER2 or puro clones were harvested by trypsinisation and expanded as cell pools.

### Immunocytochemistry, image acquisition and scoring of acini

For 2D immunofluorescence staining, cells were plated on type I rat tail collagen-coated (10 μg/ml) glass coverslips and processed as previously described.^45^ Acini were grown in eight-well culture slides (BD Falcon, Oxford, UK) and were fixed in paraformaldehyde (2% v/v final conc.), which was added directly into culture media to minimize acini loss through aspiration; staining was performed as described by Debnath *et al.*^4^ Immunodetection was performed using antibodies raised against Ki67 (#ab15580, Abcam, Cambridge, UK), HER2 (#2165S, CST, Hitchin, UK), keratin 18 Asp396 (M30 CytoDEATH mAb, #ALX-804-590-T200, Enzo Life Sciences, Exeter, UK), and Bag-1 (TB3 pAb, made in house^19^); phalloidin-TRITC (Dako, Ely, UK) was used to stain the cytoskeleton and nuclei were stained with 4'6-diamidino-2-phenylindole (Sigma). Secondary antibodies Alexa Fluor 488 goat anti-mouse IgG (#A-11001) and Alexa Fluor 546 goat anti-rabbit IgG (#A-11010) were from Life Technologies, Paisley, UK. Imaging of 2D cultures was performed with an Olympus IX81 microscope. To quantify the external morphology of acini, mounted slides were viewed using an Olympus BX51 microscope with an automated slide scanning system (Olympus Soft Imaging Systems, Munster, Germany) and high-resolution pictures covering the entire sample were captured using dotSlide v2.2 (Olympus Soft Imaging Solutions, GmbH). Images were viewed with Olympus OlyVIA v2.4 and morphology was scored by manual counting. To score acini luminal clearing or morphology, cross-sectional examination of all acini within each chamber was performed with a Leica SP5 confocal microscope; representative images were captured and analysed with LEICA LAS AF v2.6.0.

### Immunoblotting

Lysates of 2D or 3D cultures were prepared in ice-cold protease-supplemented (Sigma) radioimmunoprecipitation assay buffer (CST). Acini extracts were dissociated through a 27-gauge needle and cleared by centrifugation (13 000 r.p.m., 15 min, 4 °C). Sodium dodecyl sulfate polyacrylamide gel electrophoresis and immunoblotting were conducted according to standard protocols.^48^ Immunodetection was performed using antibodies raised against Bag-1 (#sc-56003, Santa Cruz Biotechnology, Wembley, UK), HER2, ERK1/2 (#9102, CST), P-ERK1/2 Thr202/Tyr204 (#4376, CST), AKT (#9272, CST) and P-AKT Ser473 (#4060, CST). Anti-β-actin-HRP (#3854) was from Sigma. Secondary HRP-conjugated anti-rabbit (#P0448) and anti-mouse (#P0447) immunoglobulins were from Dako. Images were acquired with a BioRad Fluor-S MultiImager using Quantity One analysis software v4.6.3. Quantification of bands was performed using the volume tools in Quantity One analysis software v4.6.6 (where a volume is the sum of the intensities of the pixels within the volume boundary, times by the pixel area). Analysis was carried out on bands pre-saturation. All bands were adjusted for global background volume. Protein abundance was calculated by normalising bands for actin and expressed relative to pcDNA.

### Cell growth assays

To measure growth, 20 000 cells/well were seeded in triplicate in 12-well plates and were fixed in ice-cold methanol on days 2 and 4 of culture. For insulin and epidermal growth factor responsiveness assays, cells which had been serum and growth factor starved for 24 h were grown in the absence or presence of the relative growth factor for 48 h and were subsequently fixed in ice-cold methanol. Fixed cells were stained with 0.1% crystal violet, rinsed in a bath of distilled water and allowed to air dry. The cell-associated dye was dissolved in 20% acetic acid and the absorbance was measured at 595 nm.

### Bag-1 inhibition

For dose–response experiments, 3000 cells/well were seeded in triplicate in 96-well plates and were treated with dimethyl sulfoxide, Thioflavin S (Sigma) or Thio-2^27^ for 5 days. Cell viability was determined by CellTitre 96 Aqueous One solution assay (Promega) according to the manufacturer's protocol. For acini morphogenesis assays, cells were pre-treated with compounds in 2D 24 h before seeding in compound-free assay media in 3D. Acini were fed with compound-containing assay media on days 4 and 8 of culture. To examine the effect of Thio-2 on signalling cell monolayers were rinsed with phosphate-buffered saline and kept for 4 h in insulin and epidermal growth factor-free media containing reduced horse serum (0.5%). Cells were subsequently treated with compounds for 1 h and lysates were prepared after stimulation with horse serum (10%) for 10 min. Unstimulated cells were used as negative controls for protein activation, whereas U0126 (Promega) was used as a positive control for inhibition of ERK activity.

### Statistical analysis

Statistical analysis was performed using GraphPad Prism version 6.00 (GraphPad Software, San Diego, CA, USA) for Windows. Analyses of more than two groups were done using two-way analysis of variance with Bonferroni's multiple comparisons test.
